# Correction: Solid-state nitrogen-doped carbon nanoparticles with tunable emission prepared by a microwave-assisted method

**DOI:** 10.1039/d2ra90002e

**Published:** 2022-01-19

**Authors:** Fitri Aulia Permatasari, Fitriyanti Nakul, Tirta Rona Mayangsari, Akfiny Hasdi Aimon, Bebeh Wahid Nuryadin, Satria Zulkarnaen Bisri, Takashi Ogi, Ferry Iskandar

**Affiliations:** Department of Physics, Faculty of Mathematics and Natural Sciences, Institut Teknologi Bandung Jalan Ganesha 10 Bandung West Java Indonesia 40132; Department of Electrical Engineering, Politeknik Negeri Batam Batam Indonesia; Department of Chemistry, Universitas Pertamina Jakarta 12220 Indonesia; Department of Physics, Faculty of Science and Technology, UIN Sunan Gunung Djati Bandung Jl. A. H. Nasution 105 Bandung Indonesia 40614; RIKEN Center for Emergent Matter Science 2-1 Hirosawa, Wako Saitama 351-0198 Japan; Chemical Engineering Program, Department of Advanced Science and Engineering, Graduate School of Advanced Science and Engineering, Hiroshima University 1-4-1 Kagamiyama Higashihiroshima 739-8527 Japan; Research Center for Nanoscience and Nanotechnology, Institut Teknologi Bandung Jalan Ganesha 10 Bandung Indonesia 40132 ferry@fi.itb.ac.id

## Abstract

Correction for ‘Solid-state nitrogen-doped carbon nanoparticles with tunable emission prepared by a microwave-assisted method’ by Fitri Aulia Permatasari *et al.*, *RSC Adv.*, 2021, **11**, 39917–39923, DOI: 10.1039/D1RA07290K.

The authors regret that affiliation *a* was incorrectly given in the original article. The correct affiliations are listed in this correction notice.

The Royal Society of Chemistry apologises for these errors and any consequent inconvenience to authors and readers.

## Supplementary Material

